# Trends in training progression: response to the study by Silkens et al

**DOI:** 10.1192/bjo.2021.1034

**Published:** 2021-11-24

**Authors:** Vivienne Curtis, Kate Lovett

**Affiliations:** South London and Maudsley NHS Trust; School of Psychiatry, HEE London; Associate Dean for Academic Psychiatry, RCPsych; Institute of Psychiatry, Psychology and Neuroscience, King's College London; and Institute of Psychiatry, Department of Medicine, University of Bolton, UK; Livewell Southwest, Plymouth; and Presidential Lead for Recruitment, RCPsych, UK

**Keywords:** Education and training, trainee choices, retention, well-being, career progression

## Abstract

Recruitment and retention are of major concern to all in medicine. Improvement in recruitment to UK speciality training programmes does not directly translate into senior workforce capacity, which remains dependent on trainee progression. In 2021, Silkens et al undertook a mixed-methods study to investigate this and described a trainee-driven shift away from conventional training pathways and expectations. These findings suggest a need for a broad change in approach to careers, underpinned by commitment to reducing differential attainment, acknowledgment that trainees may have a range of unique needs, and development of a culture of equality, diversity and inclusion.

## Background

The Royal College of Psychiatrists (RCPsych), Health Education England (HEE) and the National Health Service (NHS) have all raised concerns about the flow and retention of staff within the mental health workforce (for example, the Centre for Workforce Intelligence's review on the psychiatric workforce^1^ and HEE's *Stepping Forward* report^2^).

UK postgraduate training in psychiatry follows RCPsych curricula agreed by the General Medical Council and delivered via HEE-approved training programmes in England and by equivalent bodies in the devolved nations. There are six specialties in total (general (adult), forensic, child and adolescent, medical psychotherapy, intellectual disability, and old age psychiatry), with various dual training combinations available. All UK-based training programmes require 3 years of core psychiatric training and successful completion of RCPsych membership examinations before moving onto a separate higher training programme of 3 years (for a single specialty) or 4–5 years (for a dual higher speciality) depending on the combination of specialties. Low recruitment into core training schemes has recently been reversed by the very successful RCPsych Choose Psychiatry campaign, and recruitment into higher training is now improving. However, despite this, there continues to be significant vacancy rates within the consultant workforce. Anecdotal reports from trainees and training programme directors suggest that trainees are increasingly taking time out of training rather than completing training in the minimum time. Driven by a desire to understand this further, in 2018–2019 HEE London and the RCPsych commissioned University College London (UCL) to undertake research to further aid our understanding of career choices in psychiatry. This work formed the basis of the paper published by Silkens et al in *BJPsych Open* in 2021.^3^

## Study by Silkens et al (2021)

This was a mixed-methods project study drawing from the following data sources to understand psychiatry trainees’ career choices:

- two secondary data sources (the List of Registered Medical Practitioners and the UK Medical Education Database);
- qualitative and quantitative data from an empirical online survey;
- semi-structured interviews.

The key findings from this study were in relation to two areas, namely progression through training and experiences during it.

### Progression through training

- Psychiatry trainees overwhelmingly took longer than the 6 years anticipated to complete training.
- Training programmes have been historically designed to accommodate full-time trainees taking the minimum time to complete training. Less than full time (LTFT) trainees are often placed into whole-time posts and as a result planned absences can unhelpfully be viewed as unexpected and destabilising for a rotation.
- Progression differed between groups of trainees (direct progression for UK graduates: 18.4% *v.* non-UK graduates: 6.5%; males: 17.8% *v.* females: 12.8%).

### Experiences during training

- Satisfaction with the training programme and supervision was generally high.
- Support from peers and seniors and a sense of belonging in psychiatry were of key importance to help trainees through challenges in training.
- Positive expectations for the future were important to be able to endure high service pressure and under-resourcing; role models were very influential in shaping these expectations.
- 23.9% of trainees reported experiencing high or very high levels of burnout.
- 22% of trainees had thought about leaving the profession.
- Strong self-identity as a psychiatrist was key to keeping trainees committed to the specialty and to becoming a consultant.
- Being valued on a personal and professional level made a substantial positive difference to trainees. Without this, training was perceived as treadmill-like and impersonal.
- Trainees desired training arrangements that would support both their progression and work–life balance, including allowing out-of-programme time and LTFT hours.
- Those on longer breaks or working as specialist or associate specialist doctors were keen to become consultants. A need for more advice and support to return to training for this group of doctors was identified.

### Reflection on findings

There is a clear tension between the immediate pressures of workforce supply (i.e. to fill consultant vacancies) and supporting flexibility and choice at the expense of a slower route through training. Workforce modelling may need to adjust expectations to consider slower training with better long-term retention. While many trainees will properly continue to take the most direct route through training, where appropriate we feel that action is needed to reflect trainee experience, choices and retention.

Possible interventions could include the following.

- A *cultural change* to shift away from the idea that training takes 6 years. This does not mean normalising or accepting differential attainment, rather acknowledging, supporting and promoting trainee choices such as working LTFT and taking time out of training. Trainees must not be made to feel ‘guilty’ or ‘less than’ for taking breaks from their career. Opportunities for non-training posts (for instance, for trainees between core and higher training) could be developed to include educational activity and career mentorship, and provide different experience to that gained from a training post.
- *Proactive management of transitions* which are common and unavoidable as individuals progress in their medical careers. Some transitions may be planned, such as moving from core to higher training, but some may be unexpected, such as sickness or the need to relocate. All transitions are likely to involve challenges, but each is different and the context for individuals will vary. As clinical leaders, doctors all have to learn to manage uncertainty. Fostering the skills and attributes of psychological flexibility enables trainees to negotiate varied careers throughout their working lives while reflecting the uncertainty and difficulties their patients often face. By enabling inevitable transitions, there are opportunities to draw conscious parallels with changes in teams and services which are expected throughout the NHS and to develop leadership skills.
- Embedding employee *well-being and welfare* in clinical systems is important to recruitment and retention. Both objective and subjective perceptions of roles are critical in career decisions and may vary in relation to personal and professional situations and esteem. The UCL report highlights specific challenges in training, which mirror concerns of patients and carers in relation to under-resourcing, stigma, violence and patient suicide. The trainees in this study were at a stage where they participated in regular Balint groups, and many were from trusts with established Schwartz rounds; however, they still identified these areas of concern. Any approach which is going to improve the recruitment and retention of psychiatrists (and by implication the clinical service and patient care) needs to acknowledge the reality of clinical practice and the impact it has on staff. The RCPsych, HEE and the NHS have all developed key priorities and strategies relating to well-being. These all have a strong focus on supporting trainees identified as being in difficulty or with specific health or support needs. The NHS Staff and Learners’ Mental Wellbeing Commission report^4^ highlights 33 recommendations, including the importance of preparing for transitions, rapid access to local support, mentorship and supervision, organisational support, and culture. The RCPsych has made additional recommendations in its strategic plan^5^ and its *Supported and Valued* report.^6^

To have an impact on trainee experience and choices, stakeholders need to be engaged across national (HEE and equivalent bodies, RCPsych); regional (postgraduate deans, heads of school, RCPsych faculties and divisions) and local (medical directors, directors of medical education, training programme directors, educational supervisors, clinical supervisors, human resources and postgraduate departments) programmes.

## Conclusions

This study is a wake-up call, providing a robust description of the grassroots experience of trainees in psychiatry working in mental health services in London in 2019 prior to the Covid-19 pandemic. Since then, as psychiatrists we have all experienced unpredictable changes to the way we work and have learned to work differently, often at a distance. The lessons learned from this regarding well-being and coping with uncertainty will undoubtedly inform our future management of the workforce. However, all stakeholders need to reflect on the findings of this research project to improve the experience of training in psychiatry. Although this study focused on trainees in one specific geographical location, it is likely that there are common themes applicable at all levels of practice and localities. Safe and effective patient care demands that we employ and retain a well-trained, highly skilled, diverse and sustainable workforce. Enabling all the flexibilities needed to train and retain this generation of psychiatrists requires more sophisticated national and local workforce planning and a move away from a short-term vision of a production line.

## Data Availability

Data availability queries relating to the Silkens et al paper should be referred to the papers authors.
